# Open science, data sharing and solidarity: who benefits?

**DOI:** 10.1007/s40656-021-00468-6

**Published:** 2021-11-11

**Authors:** Ciara Staunton, Carlos Andrés Barragán, Stefano Canali, Calvin Ho, Sabina Leonelli, Matthew Mayernik, Barbara Prainsack, Ambroise Wonkham

**Affiliations:** 1 Institute for Biomedicine, Eurac Research, Bolzano, Italy; 2 Department of Science & Technology Studies (STS), University of California, Davis, USA; 3 Department of Electronics, Information and Bioengineering, Politecnico Di Milano, Milan, Italy; 4 Department of Law and Centre for Medical Ethics and Law, University of Hong Kong, Hong Kong, China; 5 Department of Sociology, Philosophy and Anthropology & Exeter Centre for the Study of the Life Sciences, University of Exeter, Exeter, UK; 6 National Center for Atmospheric Research, University Corporation for Atmospheric Research, Boulder, CO USA; 7 Department of Political Science, University of Vienna, Vienna, Austria; 8 Division of Human Genetics, Faculty of Health Sciences, University of Cape Town, Cape Town, South Africa; 9 META - Social Sciences and Humanities for Science and Technology, Politecnico Di Milano, Milan, Italy

**Keywords:** Open science, Data sharing, Governance, Benefit sharing

## Abstract

Research, innovation, and progress in the life sciences are increasingly contingent on access to large quantities of data. This is one of the key premises behind the “open science” movement and the global calls for fostering the sharing of personal data, datasets, and research results. This paper reports on the outcomes of discussions by the panel “Open science, data sharing and solidarity: who benefits?” held at the 2021 Biennial conference of the International Society for the History, Philosophy, and Social Studies of Biology (ISHPSSB), and hosted by Cold Spring Harbor Laboratory (CSHL).

## Background

Open science and the sharing of research findings, as well as research components, is emerging as a key feature of data intensive research methods. It has been credited with increasing efficiencies in research, more reproducible science, maximizing the use of a valuable resource, the democratization of knowledge (Walport & Brest, 2011), and has been credited with the rapid development of COVID-19 vaccines, therapies and diagnostics (*The Lancet*, 2021). Research participants are therefore encouraged to consent to the sharing of their genetic materials in the interest of the public, and researchers access the subsequent data (e.g. genomic sequencing data generated from such materials) to maximise their use as a valuable resource. Against this backdrop, for patients, research participants, and others to make their data available to research could seem as the obviously right thing to do: in our roles as citizens, patients, and researchers, we participate and share data. The ability to benefit from this sharing of data for research, however, is not just contingent on researchers’ access to data, but also on other contextual factors. This includes the research question(s) asked, whether social and economic equity has been a concern in the curation, use, and translation of research findings, ethical concerns around data ownership, the implications of data sharing for individuals and groups, standards and values of data quality, and the likelihood that research findings will lead to real-world changes (e.g. changes in diagnostic or therapeutic processes and instruments, etc.). Due to the asymmetry in infrastructures, resources, and capacity in data generation, storage, and analysis between researchers working in institutions in high-income countries (HICs) and low-and-middle-income countries (LMICs), questions are now being asked as to what are the benefits from this data sharing, who is benefiting, and its impact on equity. It is of key importance that open science policies pay attention to the political, economic and social factors that play such an important role in shaping who benefits from data sharing.

To discuss some of these issues, in July 2021 a plenary on “Open science, data sharing and solidarity: who benefits?” was held at the biennial conference of the International Society for the History, Philosophy, and Social Studies of Biology (ISHPSSB). The panelists (Sabina Leonelli, Ambroise Wonkham, Barbara Prainsack, Calvin Ho, Stefano Canali, and Matthew Mayernik) and chair (Katherine Littler) approached the topic from an array of perspectives. Overall, data sharing was perceived to be an important and beneficial practice. However its direction must be shifted so that it gives a more central role to equity (Nature, 2021). In this paper we report on some of the issues discussed, in particular conceptualizing open science, equity and benefit, and the governance of international data sharing for research.

### Framing open science

In reflecting on open science, the concept was framed in several different ways: it is as a core set of values to guide research, a moral standard, a project on standardization, and practically, as a way in which to overcome disciplinary silos. However it may be framed or conceived, the need for care around the language of data sharing was stressed. Comparisons between data and oil, exhaust, or other forms of corporate commodities were described as being unhelpful, and caution was urged in the use of these metaphors (Mayernik & Acker, 2018).

Discussions then turned to the implementation of open science and the sharing of data for research. It was noted that this is done through a set of common principles that include transparency, speed, reproducibility, and data quality (Wilkinson et al., 2016). While these terms may seem to be unambiguous, discrepancies arise in their application to the various different contexts in which data is collected, stored, and shared. The meaning of these principles and how they may be applied in the various contexts in which research takes place needs to be addressed as they profoundly impact research, research methods, and good research practice.

### Equity and benefit

A key focus of discussion pertained to equity and benefit in the sharing of data in research. Equity is a long-standing issue in health research, regarding ancestry research participants, the research topics, the funding allocations, workforces of researchers, and the access to publishing outlet particularly for highly expensive open access journals (Munung et al., 2018; Staunton & de Vries, 2020). This inequity has become a scientific problem in interpreting data in genomic research. The non-inclusion of enough population of African ancestry in large genomic research has resulted in at least 10% of variants missing from the reference genome. The dataset itself may also be bias, as there is limited data on populations in LMICs and marginalized populations, and this lack of diversity is preventing the equitable realization of the promise of genomics research (Rotimi & Adeyemo, 2021; Wonkam, 2021). Furthermore, researchers in LMICs often respond to funding calls that are set by funders in HICs. This is problematic as it can mean that the research agenda is not set by local research needs, but rather by the funders of research. In many of these schemes, collaboration between HICs and LMICs are encouraged with limited scope for collaboration between researchers in LIMCs within a particular region. Genomic research in Africa was cited as an example where there has been a shift in research practices in the past 10 years with some domestication of the research on the continent, but challenges remain.

A second problem related to HICs funders setting the research agenda is that they also set the conditions of funding. Generally, data sharing is required as a condition of funding. This too often serves the interests of those setting the agenda, and can end up overriding ethical concerns with personal data sharing and ownership on the ground. It also can result in a situation where there is formal formal equality in the access to data (where it is actually achieved (Bezuidenhout et al., 2017)) does not result in equity in benefit from the data. On the contrary, it can lead to a ‘Matthew effect’ whereby those who have better resources and greater capabilities are able to obtain value from research data (Merton, 1968), increasing the gap between resource-poor and research-rich areas and regions. Indeed, in the context of genomic research in Africa, published papers historically lacked local authors from where the data and samples were obtained, but served to enhance the careers of scientists in HICs (Sherman et al., 2019; Wonkam et al., 2011).

In reflecting on equity in data sharing in genomics research, panelists noted that access to the data is just one issue. Equity in the sharing of data for research is also contingent on access to the necessary technological and personnel resources to analyze and store the data, to translate findings into healthcare applications, and importantly, ensure that the local populations have access to these therapies. This echoes questions previously asked about what good is it to identify variants for breast cancer, for example, in people who won’t have access to medical treatments (Wonkam, 2021). The current system of open science needs to consider more systematically the diverse settings in which research takes place, as it rests on the assumption that all have access to the technology necessary to benefit from data sharing initiatives.

A further challenge is that the current system does not always reward the people doing most of the work (Pinel et al., 2020). The National Academies of Science 1985 Report was referenced as continuing to be of relevance today where it states that “sharing data mainly benefits society” but the “costs are born by the initial investigators” (NAS, 1985). The costs in data collection are immediate and local but the benefits often take time to accrue, are downstream and diffuse. The context in which data is shared can also impact benefit. If data is exchanged between researchers on a person-to-person basis, it is typically straightforward for the original data collector to see the downstream impacts of the secondary data use. This is much more challenging if data is accessed from a data repository, as secondary use is difficult to track and may never be known by the initial data collectors.

Finally, panelists noted that benefit is also contingent on access to good quality data. There is a need to ensure that good quality standards are maintained when the context in which they are used change, but it is difficult to apply global and general measures of quality (Leonelli, 2017). Quality can be harder to track and maintain when data is accessed from a repository without crucial information of contextual features, the diversity of research practices, environments, techniques are not acknowledged, and technology-specific and private solutions are perused. Data quality is thus closely tied to questions of metadata quality, completeness, and appropriateness for the potential data users (Rajesh et al., 2021).

With these challenges in mind, the panelists did offer some recommendations to enable equity in data sharing. Academic programs in data science and data curation must be established in universities across the world to train personnel with the necessary skills to manage, interpret, and preserve the data (Prainsack, 2019; Wonkam, 2021). They must be funded and their laboratories must have the necessary technology and infrastructure to handle and store the data. This will require the establishment of data centres across the globe. Otherwise, experienced scientists will be forced to send data overseas for analysis and storage.

There is a need to re-look at the funding of research. Research funding needs to address the critical needs discussed above to enable the equitable sharing of data in research. Equally important, data sharing requirements as a condition of funding needs to be re-examined at. It was noted that the South Africa Protection of Personal Information Act (POPIA) 2013 recently came into force and it impacts the sharing of genomic data. Genetic data is viewed as “special personal information” and its use generally prohibited unless it comes within one of the grounds of permitted use. Similar data protection regulations with similar provisions are in place in many jurisdictions across the world and may impact on the legality of some of the data sharing conditions of funding (Mascalzoni et al*.*, 2019). Panelists also pointed out that there are examples of responsible and effective data sharing, such as the Global Initiative on Sharing Influenza Data (GISAID), that should be seen as best practice in what open science should aspire to.

### Governance

Related to the issue of equity and benefit is the governance of data sharing. In this context, panellists particularly focused on where the responsibility should lie for facilitating the conversation, developing rules, and holding those involved in the sharing of data to account. Conversations on developing sustainable data practices should not just be a scientific one, but must include the public, national and international policy makers, and funders. Open science and open data are as much institutional endeavours as they are technical or scientific projects (Leonelli, 2010). It is therefore crucial that there is wide engagement across all sectors of society, as other parties may want to repurpose the data for purposes beyond its intended uses at collection. Questions were asked, however, on how best to develop practices that reflect a diversity of views both across the differing stakeholders, but also across the differing contexts in which science takes place, while also allow science to continue and progress. The role of geopolitics in this conversation was acknowledged. This may be due to scientific competition between different nations, but equally could be due to the need to protect data as a resource. As one example, caution was urged about the need to prevent a “gold rush” for African genomic data. Considering this geopolitical climate, an honest broker was called for to drive forward a collective agenda in data system.

Specific legislation on open science was called for, as well as the need for national and international governance to stop the exploitation of data. Open science was described as an instrument of neo-liberalism that is reflective of an Anglo-American narrative. This is in part due to the fact that open science has been shaped by agendas set in HICs with little or no consideration of other contexts (Maxson Jones et al., 2018). It was suggested that a federated system of governance that is participatory in approach could change the narrative. A federated system enables legal data control to remain within the country from which the data originates and the Global Alliance for Genomics and Health (GA4H) was cited as a good example (GA4H, 2016). What is important is that in the development of standards and governance, a contextual approach is taken, that is responsive to the different contexts in which data takes place, the different repositories in which data is held, the different relationships in which data is exchanged, and the different type of data that is shared (Canali, 2020).

## Conclusion

Data-intensive methods are transforming every aspect of biological and biomedical research, but changes are required so that it develops in a more equitable manner, in data generation, access, analysis capacity and intellectual property regimes. This year (2021) marks the 25th anniversary of the Bermuda Principles on DNA Sequence and Data Sharing (1996). Research adherence to the accord shows mixed balances: more success around the sharing aspect of it in some parts of the world and less success on delivering benefits to specific communities. A critical investigation of who benefits is fundamental to the entire enterprise and in moving forward, consideration of equity must be central. Conversations on how to achieve this, must be free of the current narrative that primarily rewards those in HICs who have set the agenda. Despite the problems that are inherent in the current system, there are many examples of good and responsible practices in data sharing. Just as organisations like the Research Data Alliance, CODATA and World Data Systems have been advocating (CODATA, 2020), these examples must be examined, and used to inform governance models for equitable data sharing.
